# Review of Microfluidic Methods for Cellular Lysis

**DOI:** 10.3390/mi12050498

**Published:** 2021-04-28

**Authors:** Emil Grigorov, Boris Kirov, Marin B. Marinov, Vassil Galabov

**Affiliations:** 1 Faculty of German Engineering Education and Industrial Management (FDIBA), Technical University of Sofia, 1756 Sofia, Bulgaria; vtg@tu-sofia.bg; 2 Department Industrial Automation, Technical University of Sofia, 1756 Sofia, Bulgaria; boris.kirov@tu-sofia.bg; 3 Department of Electronics, Technical University of Sofia, 1756 Sofia, Bulgaria

**Keywords:** cell lysis, cell membrane, microfluidics, review

## Abstract

Cell lysis is a process in which the outer cell membrane is broken to release intracellular constituents in a way that important information about the DNA or RNA of an organism can be obtained. This article is a thorough review of reported methods for the achievement of effective cellular boundaries disintegration, together with their technological peculiarities and instrumental requirements. The different approaches are summarized in six categories: chemical, mechanical, electrical methods, thermal, laser, and other lysis methods. Based on the results derived from each of the investigated reports, we outline the advantages and disadvantages of those techniques. Although the choice of a suitable method is highly dependent on the particular requirements of the specific scientific problem, we conclude with a concise table where the benefits of every approach are compared, based on criteria such as cost, efficiency, and difficulty.

## 1. Introduction

Cells are the fundamental unit of all living organisms. The genetic information for the development and functioning of any organism is encoded in their DNA or RNA sequences that are located inside the cell [1]. Every cell has an outer boundary called a cell membrane, which serves as a barrier and regulates the transport across the cellular membrane of material between the inside and the outside of the cell. Cell lysis is the method through which this outer cell membrane is broken down to release intracellular substances, such as DNA (deoxyribonucleic acid), RNA (ribonucleic acid), protein, or organelles. The process is an important and necessary part of the molecular diagnostics of pathogens, protein purification, disease diagnostics, drug screening, mRNA (messenger RNA) transcriptome determination, and analysis of the structure of specific proteins, lipids, and nucleic acids. To determine lysis efficiency, quantitative real-time polymerase chain reaction (RT-qPCR) should be evaluated. There are several methods, which are commonly used for cell lysis, including mechanical disruption, liquid homogenization, high-frequency sound waves, and reagent-based (chemical) methods. In most cases, these processes are very demanding and time-consuming, despite the development of today’s technologies [2]. Scientists are therefore constantly searching for simple alternatives to conventional expensive laboratory equipment. One possible way of making cell lysis more efficient and quick are the so-called microfluidics devices [2,3]. These devices make it possible for a tiny amount of liquid to be handled and visualized, providing the possibility for high efficiency, easy operation, and low production costs for the conducted processes [1]. Due to the low volumes required, microfluidic technologies represent a promising alternative to conventional laboratory techniques. Their application is often associated with decreased sample and reagent consumption, shorter experiment times, and reduced overall costs of the setup. In their article, Nandagopal et al. compared the efficiencies of a microfluidic and a conventional method for the lysis of *Escherichia coli* (*E-coli*) cells, utilizing CuO nanoparticles (mechanical lysis) [4]. The results showed an increased efficiency by more than 30% when using the channels of the microfluidic chip. This was due to the high surface area to volume ratio and active internal circulation in microfluidic flows. The main limitations associated with microfluidic devices are the requirement of external pumps, tubing, and connectors, making the system somewhat dependent on those consumables. Microfluidic devices are usually not rugged, which may cause flow profile problems due to leakage or uneven pressure in the channels [5].

This review will focus on the cell lysis methods utilized in microfluidics on-chip devices for cell analysis. The adoption of a particular method depends very much on the specific concrete application and project limitations of the problem. Finally, we will deliver an objective statement about the advantages and disadvantages of the individual methods.

## 2. Cell Lysis Methods

Numerous methods and possibilities for microfluidics cell lysis are reported in the literature in recent years [2,6]. The dissimilarities between them are due to the variety of existing physical effects used to rupture the cell membrane. This leads, in most cases, to different efficiency, speed, technical complexity, and costs of the process [6]. In the following, the five most commonly used methods (chemical, mechanical, thermal, electrical methods, and laser lysis) are described using various scientific articles.

### 2.1. Chemical Lysis

**Figure 1:**
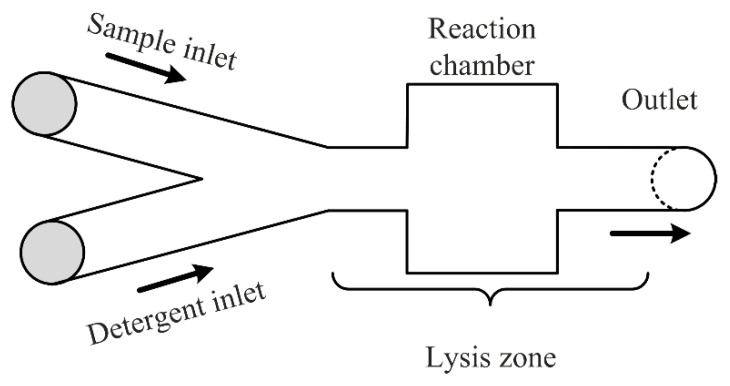
Schematic generalization of a microfluidic device for chemical lysis.

Chemical methods make use of lysis buffers to disrupt the cell membrane. Detergents can also be added to cell lysis buffers to solubilize the membrane proteins and rupture the cell membrane to release its contents [2,7]. Chemical lysis can be classified as alkaline and detergent lysis [2]. In the first type, OH− ions are the main component utilized for lysing target cells. The method is suitable for all kinds of cells, the process is however very slow and can take up to 12 h. The second type of chemical lysis utilizes detergents (also called surfactants) to disrupt the cell membrane. Detergents are most widely employed for lysing mammalian cells. For lysing bacterial cells, however, where multiple layers enclose the cell content, first, the outer cell wall has to be broken down to rupture the cell membrane [8]. In most cases, detergents are therefore utilized along with lysozymes for lysing those types of cells. Generally milder non-ionic detergents are preferred as they cause the least amount of damage to proteins and enzymes, for example, zwitterionic detergent and Triton-X [9]. Chemical lysis is the most utilized method in the microfluidic world, because of its simplicity and uncomplicated way of handling it. Figure 1 shows a generalized schema of the method. In general, separate inlets (one for the sample flow and one for the detergent agent) with one or two syringe pumps and a common outlet are necessary. A reaction chamber may be useful to visualize and measure the lysis efficiency better. Due to the laminar characteristics of the flows in microchannels (low Reynolds numbers), one should always take into account the possibility of poor mixing between the two fluids and therefore consider adding geometry constructions (passive mixing methods) to the channels [10].

Several papers concentrate their work on comparing the lysis efficiency of different lysis buffers and detergents. For example, Chen et al. compared the lysing percentage and lysing efficiency of guanidine and Triton X-100 lysis buffer, utilizing a sandwich-type microfluidic device for rat blood cell lysis [11,12]. Based on the dimensions of the microchannel, it was figured out that the time taken for complete blood cell lysis by guanidine salt was 174 s, by Triton X-100 161 s, and by mixture buffer C 108 s. The experimental results showed also that an increased cell flow rate leads to shortening of the flow time of the solution, therefore reducing the chances of exposing cells to the lysis buffer, thus leading to worse cell lysis. To evaluate the degree of mixing at any location in the microchannel, a mixing index (*σ*) from [13] was utilized:

(1) $$\sigma =\left(\frac{1-\int_{h}^{b}\left(c-c_{a}\right)dy}{1-\int_{h}^{b}\left(c-c_{\infty }\right)dy}\right)\times 100\%$$

*c* is the species concentration profile across the width of the microchannel with the length h in the y-direction, *c*_∞_ the completely mixed state (=0.5), and *c*_0_ is the completely unmixed state (=0 or 1).

**Figure 2:**
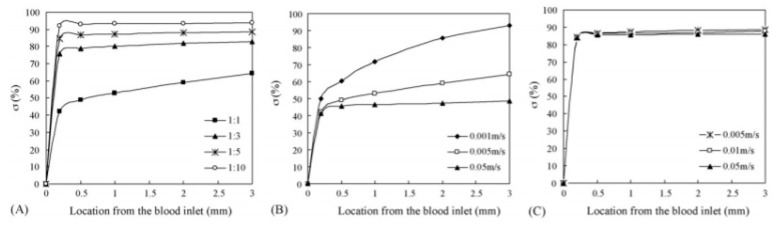
Numerical evaluations of *σ* at a variety of flow conditions are shown in [12]. (**A**) The flow rate of blood was 0.005 m s^−1^, whereas the flow rate of the lysis buffer was 0.015, 0.025, 0.005, and 0.05 m s^−1^. (**B**) The ratio of the blood’s flow rate to the lysis buffer was 1:1. (**C**) The ratio of the blood flow rate to the lysis buffer was 1:5. Adapted with permission from [12]. Copyright 2007 Copyright Elsevier.

The numerical results for *σ* are shown in Figure 2. The relationship of σ and the flow ratio asserts lower blood/lysis buffer ratio leads to better mixing. The flow rate has a negative effect on σ when the ratio is 1:1. At a ratio of 1:5, the mixing index does not change with increasing flow rate. Under the condition of higher flow rate and lower ratio, rapid mixing can be implemented.

**Figure 3:**
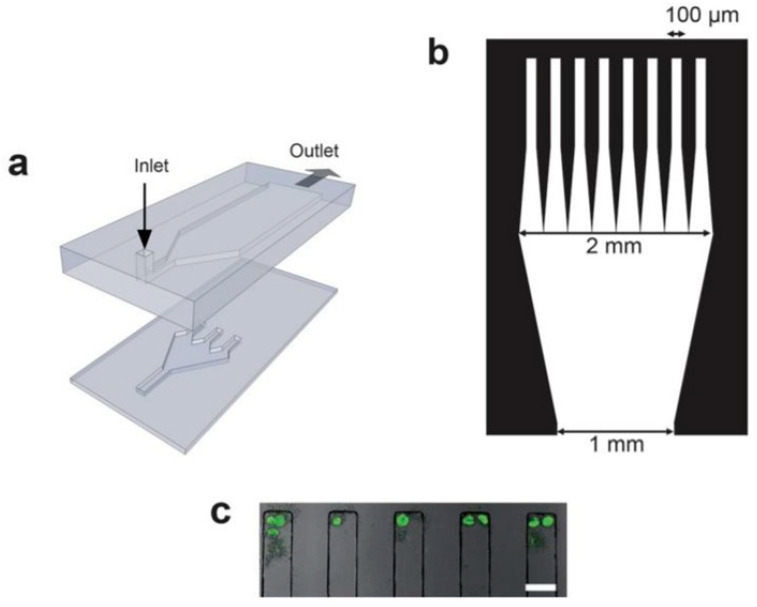
(**a**) Microfluidic device in which two PDMS channels facing each other are sealed together [14]. (**b**) Diagram of the chambers along with the channel dimensions. (**c**) Image of Arcella incubated with fluorescein diacetate in capture chambers. Adapted with permission from [14]. Copyright 2009 Copyright Royal Society of Chemistry.

Similarly, Heien et al. reported a microfluidic device with a series of chambers, capturing cells and preventing them from moving while under continuous flow [14]. With this device, the lysing efficiencies of benzalkonium chloride, chlorhexidine digluconate, phenol, sodium dodecyl sulfate (SDS), and Triton X-100 buffers were compared–Figure 3. Differences in lysis and decay times for Arcella Vulgaris cells were observed at different flow rates and concentrations of benzalkonium chloride. At higher flow rates, more detergent is delivered to cells in the device, whereas at lower flow rates, less detergent is delivered and there were longer lysis decay times. Since Arcella is an amoeboid species, it contains a negatively charged membrane that hinders lysis by SDS; thus, neither SDS nor phenol induced lysis after 40 min of exposure [15]. For the same reason, benzalkonium chloride was an effective lysis agent of Arcella Vulgaris as it was a cationic detergent, enabling the effective solubilization of the cell membrane components. Triton X-100, a nonionic detergent, was similarly effective in causing lysis, characterized by short decay and lysis on-set times. A comprehensive list of detergents can be found in [16].

**Figure 4:**
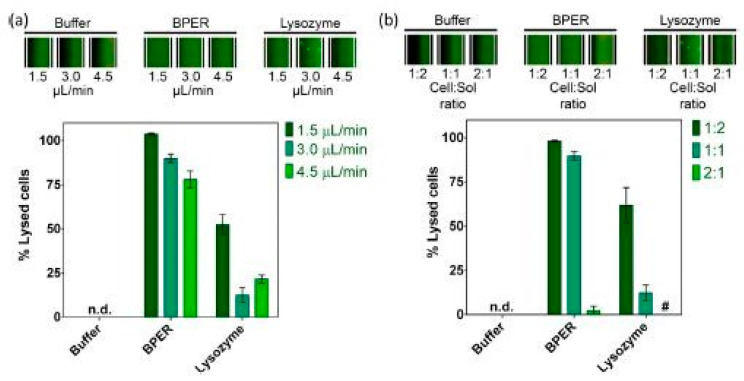
Percentage of cell lysis obtained in [18] for buffer resuspended cells, using Tris–HCl buffer, BPER, and lysozyme. (**a**) For different total flow rates (using the same flow rates of resuspended cell solution and buffer or lysis solution); (**b**) for different flow rate ratios (keeping constant total flow rate). Adapted with permission from [18]. Copyright 2020 Copyright Elsevier.

Shamloo et al. described the process of chemical lysing of mammalian cells within a droplet-based microfluidic chip, utilizing a computational fluid dynamics (CFD) model [17]. Both cell solution and lysis reagents were encapsulated within a droplet, the lysis procedure was simulated inside the droplet. It was shown that even a significant increase in the concentration of the lysis reagent does not increase the lysis efficiency of the cell solution. On the other hand, increasing the volume fraction of the lysis reagent (the ratio of the volume initially filled with the lysis reagent to the total volume of the droplet) to 97%, achieved a complete cell solution lysis within 0.25 s. R. Fradique et al. reported a diffusion-based microfluidic device for the rapid screening of continuous lysis conditions, utilizing *E. coli* as a model host [18]. The main goal of the work was to determine the efficiency of different lysis conditions for the release of an intracellular protein from the bacteria cells, in other words, to measure the effect of different lysis solutions, contact times, and cell to lysis solution volume ratios. The results are shown in Figure 4. A commercial BPER solution, based on non-ionic surfactants, and an enzymatic solution of lysozyme were utilized. The commercial solution showed, even for the highest flow rates tested, lysis efficiencies above 75%, meaning that it could be utilized for higher throughput microfluidic systems, where rapid cell lysis is required. Similar results were shown in [19].

**Figure 5:**
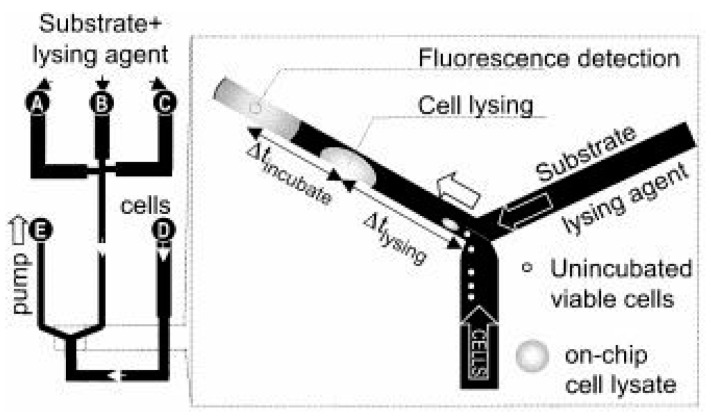
Scheme of the on-chip lysing and on-chip incubation of HL-60 cell with substrate developed in [20]. A fluorescence detector was located downstream of the mixing point, and an observation microscope sat over the intersection. Adapted with permission from [20]. Copyright 2004 Copyright IEEE.

Gregor Ocvirk et al. described a typical example of a conventional chemical cell lysis device [20]. A Y-shaped microfluidic chip for mixing cell steam (physiological buffer) with a lysis buffer stream (Triton X-100) was developed. The cells were lysed within 30 s. Figure 5 shows the constructed device and the mixing area.

Mahalanabis et al. reported a hybrid chemical/mechanical method for the lysis of Gram-positive and Gram-negative bacteria on a microfluidic chip. The authors utilized a silica porous polymer monolith in the presence of a lysis agent (sodium dodecyl sulfate (SDS) and TritonX-100 at different concentrations), to produce additional shear and friction forces to the pressure-driven solution flow [21]. The lysis was confirmed by the successful isolation of PCR quality bacterial DNA from both Gram-negative (*Escherichia coli*) and Gram-positive (*Bacillus subtilis*) bacteria. Because of the extensively cross-linked peptidoglycan layer in Gram-positive cells, the PCR threshold cycle (Ct) at which the target DNA was amplified was much lower, compared to negative-gram cells. Overall, the device worked comparably to the standard benchtop bacterial and viral lysis and nucleic acid extraction kits. This technique was also implemented by Mirer et al. for the design of a complete microTAS for detecting bacteria [22]. To further increase the lysis efficiency and the amount of amplified DNA when dealing with Gram-positive bacteria cells, Lui et al. proposed a microfluidic chip for single-cell whole-genome sequencing (SC-WHS) [23]. By combining thermal treatment (heat-shock) with chemical lysis (alkaline-based buffer), 100% of the bacterial single-cell lysis rate was achieved. Corynebacterium glutamicum and Nostoc sp. were chosen as a Gram-positive and a Gram-negative model respectively, due to the significant lysis difficulties encountered in previous studies [24] and [25]. The results showed also that all three species reached a 100% single-cell amplification rate and an average of 66.5 ng, 73 ng, and 42.8 ng of DNA respectively.

**Figure 6:**
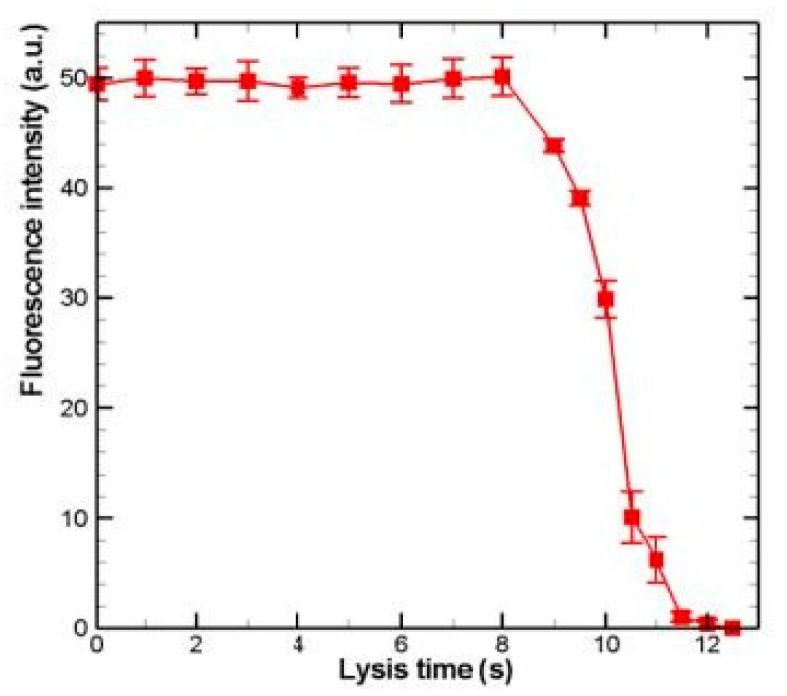
Fluorescence intensity of a single HeLa cell versus the time after the introduction of the lysis buffer shown in [28].

Another example of a chemical lysis technique is given in [26]. The author’s approach to the chemical lysis of single cells involves a microfabricated device that handles and mixes 25 pL of two fluids, using a cell crossover (CCO) technology. The first fluid is the single-cell carrier and the other the lysing solution. When cells pass through the CCO region, where they move from the carrier solution to the lysis solution, they are lysed immediately. The device was proven to have 86% of the capacity of the conventional chemical lysis method based on a comparison of the extracted DNA quantity. Although the aforementioned device can effectively lyse cells, it is difficult to analyze and observe the single-cell lysis process in detail, according to the authors. To do this, Daniel Irimia et al. developed a new lysis method [27]. Cells and fluids are independently isolated in two chambers using the geometry of the microchannels and the four billed thermo-pneumatic actuators. When cell solution flows through the upper lysis chamber, the dam-like structure captures a single cell. Then, by removing air from the mixing channel, lysis solution and cell solution are mixed to complete the cell lysis. Following this step, a stable dilution of intracellular components is achieved. According to the authors, the usage of the device can help for the direct estimation of the intracellular concentration of a soluble dye and the indirect evaluation of intracellular quantities of insoluble actin. To further increase the lysis efficiency at a single-cell level, Jen et al. developed a microfluidic chip, which utilized a dense array of microwells to trap the cells (HeLa cells) [28]. Two different sizes of the microwells were compared. The cellular occupancy in 30-µm-diameter-diameter microwells (91.45%) was higher than that in 20-µm-diameter-diameter microwells (83.19%) at an injection flow rate of the sample of 2.8 L/min. On the other hand, most of the occupied 20-µm-diameter microwells contained individual cells. A detergent-based buffer was then introduced to the channels, resulting in the chemical lysis of the trapped cells. The fluorescent intensity of the cells was then measured. The results are shown in Figure 6, with the data based on measurements in at least three individual cells, the points representing the average value. The intensity drops dramatically at 8 s, reaching almost zero at 12 s.

**Figure 7:**
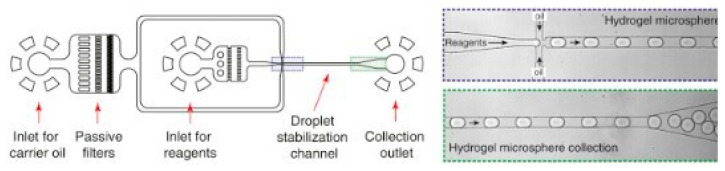
The microfluidic platform utilized in [33] encapsulates cells into droplets with lysis buffer for cell lysis.

Yasuhiro Sasuga et al. reported a microfluidic lysis chip that utilizes microchannels, where cell solution and lysis buffer are injected successively by a syringe pump [29]. The cells are deposited in the microwells, thus allowing the single-cell lysis to be examined in detail through inverted fluorescence lightning. The results of the experiments indicate that cell membranes were gradually lysed as the lysis buffer was injected in a period as short as 12 s. In [30], a novel microfluidic platform was demonstrated by SoHoo et al., which gave a better look at the kinetics of chemical lysis. With 20 different observation points over 23 mm, travel times through the device, the effect of the lysis reagent on the cells, lysis concentrations, and rates were measured. The minimum lysis reagent concentration needed to initiate lysis was found to be less than 1%. At this concentration, lysis was initiated in 0.2 s. This implies that using a significantly lower concentration than the standard amount of lysis solution is possible but will result in longer lysis times. The results of the rate analysis showed that increasing the concentration of the lysis reagent does not increase the lysis rate much beyond 10%. Chemical lysis, as described above, is the most commonly utilized method in droplet-based microfluidic chips due to the efficient penetration of the membrane components [31]. Ramji et al. described a droplet-based microfluidic chip, which enables controlled exposure of isolated single cells to a high pH buffer [32]. The device comprises a cell-focusing channel, a reagent injection inlet, and a droplet generator for single-cell lysis. Individual mammalian cells are encapsulated into the droplets with chemical reagents, which results in disrupting the cell membrane integrity, causing cell lysis. Figure 7 shows an example of a droplet generation combined with chemical lysis in a microfluidic device developed by Klein et al. in [33]. The platform encapsulated cells into droplets filled with lysis buffer. Similar droplet-based microfluidic devices, utilizing chemical lysis detergents are described in [34,35,36].

### 2.2. Mechanical Lysis

**Figure 8:**
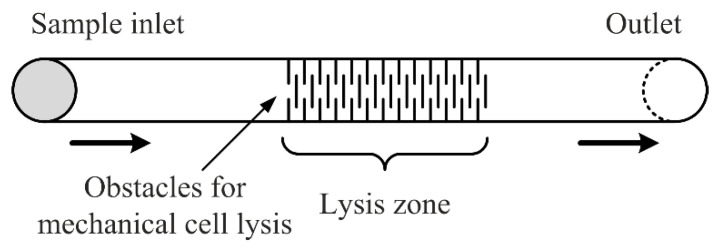
Schematic generalization of a microfluidic device for mechanical lysis.

In mechanical lysis, the cell membrane is physically broken down by using sheer force. The technique involves directly damaging the cell structure to release the required intracellular components [2]. The most commonly used technique for mechanical lysis is the integration of small nanoscaled-obstacles in the microchannels, which eventually squeeze the cells and destroy their walls. Figure 8 shows a generalized schema of the method. Here, one inlet with one syringe pump is enough, as there are no other fluids needed for the process, except for the sample. Before constructing the microfluidic device, one should always make sure that the induced shear stress from the designed obstacles will be high enough to destroy the cell walls. It is also possible to make use of capillary effects (i.e., capillary pump) in the lysis region, to accelerate the flow and reach higher shear forces [37].

**Figure 9:**
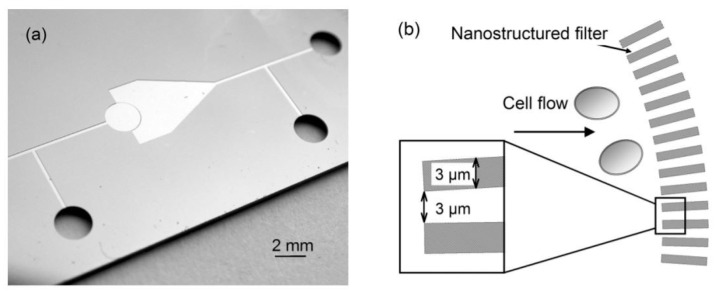
(**a**) The nanostructured mechanical filter utilized in [38]. (**b**) A schematic of the mechanical lysis portion of the device Adapted with permission from [38]. Copyright 2003 Royal Society of Chemistry.

Dino Di Carlo et al. developed a mechanical lysis device integrated with additional sharp nanostructures in the channels, concentrating and amplifying fiction forces to penetrate the cell membrane [38]. Figure 9 shows the device and its functionality, which proved to be simple and effective and caused no harm to the extracted proteins. The nanostructured barbs on the walls increased the accessibility to proteins by three times, compared to filters without the sharp structures.

**Figure 10:**
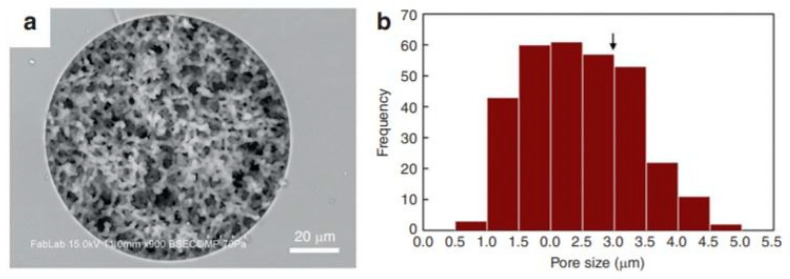
(**a**) Image of the silica monolith utilized in [39] for mechanical cell lysis. (**b**) Histogram of pore size. Critical diameter for RBC hemolysis is marked with an arrow.

On the other hand, Han et al. utilized the rigid walls of bacteria cells to develop a microfluidic device with integrated porous silica monoliths. Monoliths are highly porous materials presenting tortuous fluid flow paths, therefore inducing high-mechanical surface stress during cell perfusion, enabling mechanical lysis of blood cells, and allowing intact and viable bacteria to traverse the porous flow paths. Figure 10 shows a picture of the monolith itself and the frequency of the pore sizes, 1.5–2.5 μm being the most frequent sizes. The isolation of intact and viable Gram-positive and Gram-negative cells from whole blood was successfully achieved through selective lysis due to the differences in diameters (erythrocytes at 3–6 µm and bacteria at 0.8–2 µm) and membrane tension between bacteria and blood cells. The serial passage through multiple monoliths lysing allowed the destruction of 99.9% of the red blood cell membranes and nearly 100% of bacteria to be recovered. By isolating the bacteria within defined locations of the filtration device, the selective lysis provided an effective approach to sample preparation for downstream analysis of bacteria cells [39].

**Figure 11:**
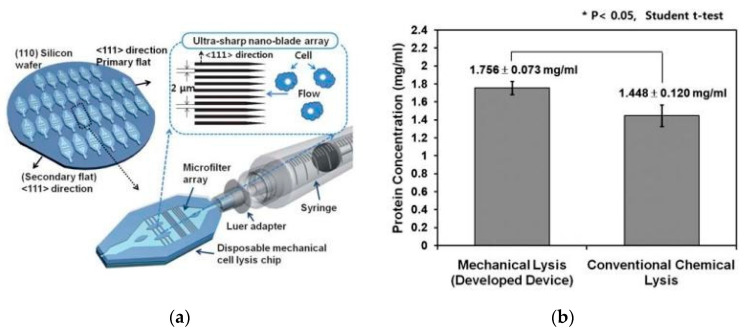
(**a**) Schematics of the chip for mechanical cell lysis chip with ultra-sharp nano-blade arrays utilized [40]. (**b**) Comparison of protein concentration between the developed mechanical cell lysis method and the conventional chemical lysis method. Adapted with permission from [40]. Copyright 2010 Royal Society of Chemistry.

A handheld microfluidics chip with ultra-sharp nano-blade arrays [40] was presented, based on a similar method by Sung-Sik Yun et al. Figure 11 shows the working principle of the device and a comparison of the protein concentration to the one prepared by a conventional chemical lysis method. The total lysis times required for the mechanical cell lysis and the conventional chemical lysis were less than 2 and 30 min, respectively. The developed design showed an 18% higher protein concentration, compared to the chemical lysis.

**Figure 12:**
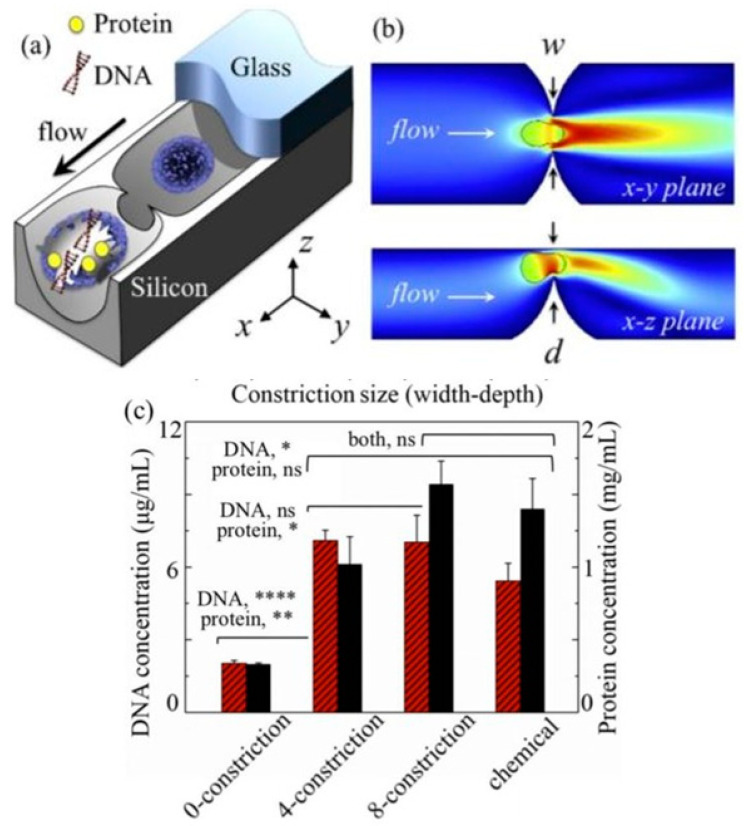
Single-cell point constriction utilized in [41] for reagent-free cell lysis. (**a**) Cell being ruptured by the ultra-sharp edge of a round constriction. (**b**) A cell undergoing excessive rapid deformation through a point constriction. (**c**) Comparison between the results of lysates obtained from a device without any constriction or from a conventional chemical method.

Sharp nanostructures built into the flow path have been pursued by several other research teams for reagent-free cell lysis. Huang et al. reported a silicon-glass microfluidic device featuring point constrictions for reagent-free mechanical cell lysis and intact nuclei isolation on a single-cell [41]. Because of the characteristic geometry (concave nanoblade), single-cell constrictions were conducive to isolating intact nuclei. Mouse embryo fibroblasts (NIH/3T3) and human colorectal carcinoma cells (HCT116) were utilized for the measurements. The results from a conventional chemical lysis treatment on the identical design of the chip, but without any constriction, were compared to the results from the mechanical structure. The measurements suggested that the size and count of constrictions must be tailored according to a specific cell type for the maximum recovery of total proteins. A device with four- or eight-constriction treatment groups exceeded the average value of the chemical group for the number of lysed cells, as shown in Figure 12. Similar obstacle-based microfluidic devices, utilizing mechanical lysis to rupture cell membranes, are described in [42,43,44].

It is known that just subjecting cells to frictional forces induced by cell contractions may not always be enough to rupture cell membranes [45]. Therefore other types of mechanical lysis methods have been developed. Yu Chang Kim et al. reported a new kind of microfluidics biomechanical device for compressive stimulation and lysis of cells [46]. Compressive stress is directly applied to the cells through a deflected polymer membrane between two microchannels. To monitor cell deformation and lysis with the applied pressure, MCF7 cells were compressed at an elevated pressure ranging from 0 to 50 kPa for loading membrane. The cell was gradually ruptured by the applied compressive force. With the resulting pressure, the membrane deflection increased and small bulges appeared at the cell membrane. The cell lysis was assumed to be carried out within 135 ms ± 37 ms for MCF7 cells. In other words, the cells were mechanically burst and lysed at 35 kPa through the compressive force.

**Figure 13:**
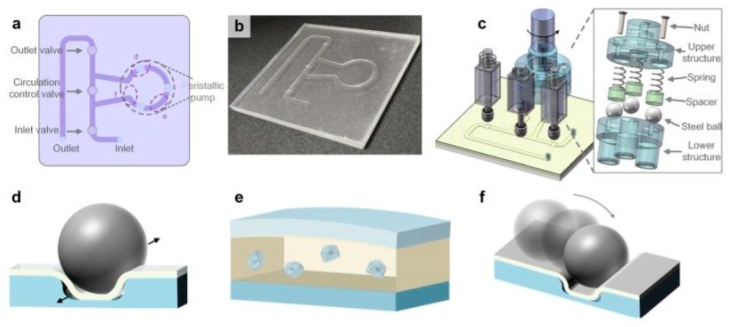
The pump-on-chip cell disruption microfluidic chip utilized in [50]; (**a**) schematic of the device, (**b**) picture of the fabricated cell disruption microfluidic chip. (**c**) Schematic of the platform with the on-chip micropump, electromagnets. (**d**) Cells in the sample leaking through the gap between the channel corners and the PDMS membrane are pulverized by the steel balls. (**e**) The compressive stress makes cells deform. (**f**) Some cells are crushed down by the steel balls. Adapted with permission from [50]. Copyright 2017 AIP Publishing.

More efficient microfluidics systems for cell lysis, based on a CD-like structure were described in [47] and [48]. When the entire disk spins, the induced rotating magnetic field drives ferromagnetic blades. Through the relative motion of the ferromagnetic blades and grinding matrices, cells are ruptured by mechanical impact and shear forces. Other techniques utilized for the mechanical distribution of cell membranes are the bead-beating and sonication methods. Vandeventer et al. [49] mentioned in their work that these mechanical methods were more efficient to utilize for the disruption of lysis-resistant bacterial cells such as *Bacillus anthracis* spores and *Mycobacterium tuberculosis* cells. Conventionally, these techniques use a crushing action to disrupt the bacteria and spore cell walls. A typical example of a bead-beating mechanical lysis method is shown by Cheng et al. in [50]. Figure 13 shows the developed microfluidic platform, based on an on-chip micropump for mechanical cell disruption. The rotary motor of the pump contained three electromagnets for valve control. On the motor head, three steel balls were evenly mounted and were set in contact with the annular channel to disrupt cells and pump fluid. The driving device of the micropump consists of a lower structure, steel ball, spacer, spring, nut, and upper structure. The influence of the pressing depth of the steel balls on the chip on cell disruption performance was evaluated. The cell disruption rate first increased with the pressing depth, achieving the maximum of 56.5% at 250 pm. The effect of the motor rotation speed on cell disruption was also evaluated by pumping the 50 µl sample with 9 different rotation speeds. The cell disruption rate increased slowly with rotation speed and achieved 56.2% at 112 rpm. Similar lysis efficiency levels by the bead-beating method were shown in other papers as well [51,52,53,54].

Flaender et al. showed a semi-automated operation of grinding lysis on a microfluidic device [55]. The lysis was performed by grinding the targets against a frosted glass, using a spatula. In the manual method, approximately 30 s were required for full injection of a 1 mL sample. The scraping motion of the spatula during approximately 30 s was sufficient to lyse the spores/bacteria (*Bacillus subtilis* endospores, *Bacillus globigii* endospores, *Escherichia coli*, and *Bacillus globigii* bacteria).

**Figure 14:**
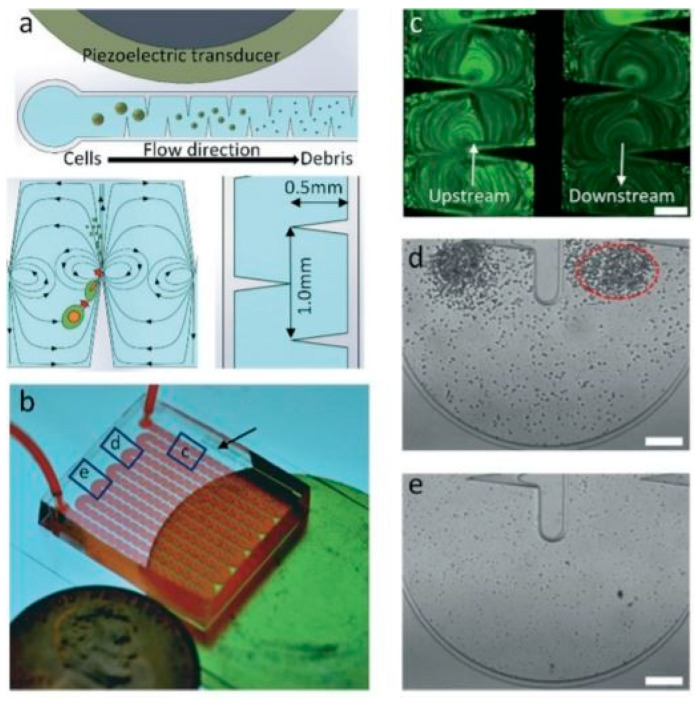
Design and concept of the sharp-edge-based acoustofluidic device for cell lysis utilized in [56]. (**a**) Schematic overview of the mechanism of the acoustofluidic lysis device. Sharp-edged structures are constructed on the sidewalls of the channels. (**b**) The device is composed of a serpentine channel with a large number of sharp-edged structures, an acoustic transducer, and a thin glass substrate. (**c**) Comparison of the cell density downstream and upstream of the channel. Image (**d**) upstream and (**e**) downstream the channel. Adapted with permission from [56]. Copyright 2019 Royal Society of Chemistry.

The demonstrated results showed that the portable and very low-cost manual mechanical lysis device allows the detection of as low as 5–10 spores/mL in 250 pm samples. It was also possible to process larger volumes of the sample (3–10 µL) by the improvement of the filling system. Wang et al. demonstrated a novel method of an acoustofluidic device for the lysis of HeLa and Jurkat cells [56]. The technique introduced an acoustic radiation force and acoustic derived streaming in a microfluidic system. In the lysis device, cells were primarily lysed mechanically by the shear forces that arise from the fluid motion induced by acoustically oscillating sharp-edged structures. By controlling the strength of the driving voltage the magnitude of the resulting shear forces can be controlled by adjusting the strength of the induced acoustic streaming effect), the device is shown in Figure 14. Lysis efficiencies higher than 90% (98% in some test runs) were achieved.

### 2.3. Electrical Methods

**Figure 15:**
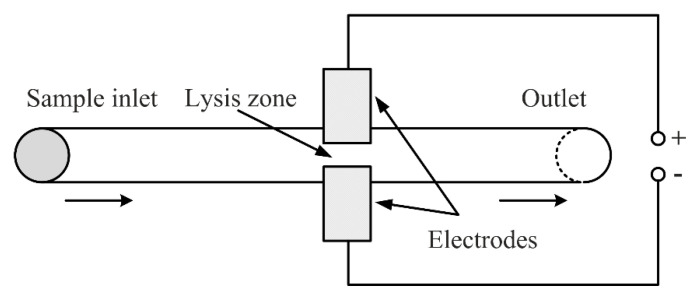
Schematic generalization of a microfluidic device for mechanical lysis.

Electrical methods (also called electroporation) for cell lysis are another common method for creating transient pores in cell membranes. A potential across the cell membrane is being created, known as the transmembrane potential (TMP) when exposing cells to an external electric field. Once the potential exceeds a certain threshold, pores form on the membrane to release intracellular components. Unlike the detergents utilized in chemical lysis, it is known that there are no effects on the intracellular contents after application of an electric field high 4 [57,58]. Electroporation-based devices do not differ from mechanical- and chemical-based devices regarding their flow drive mechanisms. The electrodes usually are made of Gold [59], Platinum wire [60] but also other materials. Fox et al. made a summary of the electrode potentials needed for the lysis of certain types of cells [61]. Figure 15 shows a generalized schematic of an electroporation microfluidic device for cell lysis.

**Figure 16:**
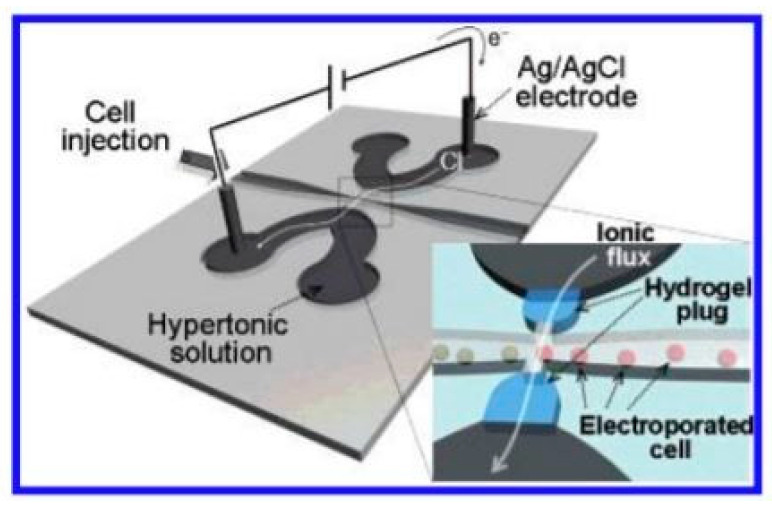
The schematic of the micro-electroporation chip is utilized in [65]. Cells in the region between the salt bridges experience an electric field. Adapted with permission from [65]. Copyright 2007 American Chemical Society.

Using a direct current (DC) electric field is normally being associated with the largest possible TMP. However, it produces also a voltage exceeding the water electrolysis threshold (1.3 V), which may inevitably create gas bubbles and extreme pH conditions near the electrodes [62]. For this reason, the usage of alternating current (AC) electric field for lysis was investigated in [63]. The group finally concluded that at frequencies higher than 0.15 Hz no bubble formation occurs at any of the tested flow rates (3 to 300 µl per min). Roee Ziv et al. designed also a microchip based on AC electric field with liquid electrodes [64]. By regulating the voltage and frequency, cell membranes could be optimally broken down. The problem of gas bubbles was also solved by Sang Kyung Kim et al. by a microfluidics chip with polyelectrolyte salt bridges [65]. A pair of plugs on both sides of the microchannel separate the cell suspension and a hypertonic solution. The ionic flux completes the electric circuit from an external electrode to the other electrode. The chip was designed to have low impedance so that a large portion of the potential difference was exerted on the cell flux, achieving a sufficiently high electric field gradient by a small DC bias and avoiding which avoids gas bubble formation. Figure 16 shows the experimental setup.

**Figure 17:**
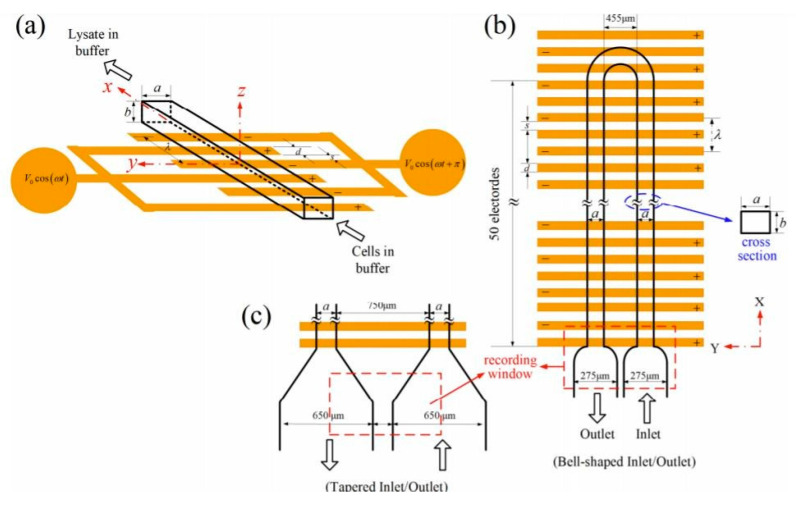
(**a**) Sketch of a simple flow-through device for continuous electrical lysis of cells utilized in [67]. (**b**) Top view of the device. (**c**) An alternative inlet/outlet design for reducing the flow in comparison with that in (**b**), for a clear observation of cell lysis utilized in [67]. Cells in the region between the salt bridges experience an electric field.

Xiao-Yu Wei et al. designed a fully transparent microfluidic chip device for the lysis of individual cells with built-in transparent ITO electrodes [66]. Cells were successfully lysed under low-voltage conditions (16 V peak-to-peak or ±8 V), which provided a safe, reliable, continuous, and controllable cell lysis process. In comparison with physical and chemical cell lysis, a higher voltage and an optimized frequency could increase the speed and efficiency of the lysis. A similar device was presented by Lo et al. [67]. It consisted of a rectangular microchannel with a planar electrode built on its bottom wall, actuated by alternating current (AC) voltages between neighboring electrodes, as shown in Figure 17. Human whole blood was pumped through the device; the cells were completely lysed within 7 s after the application of a 20 V peak-to-peak voltage at 1 MHz, and volume flow of up to 400 µLh−1. The results showed also that only the lower half-channel was exposed to an electric field exceeding the irreversible threshold value of cell electroporation.

**Figure 18:**
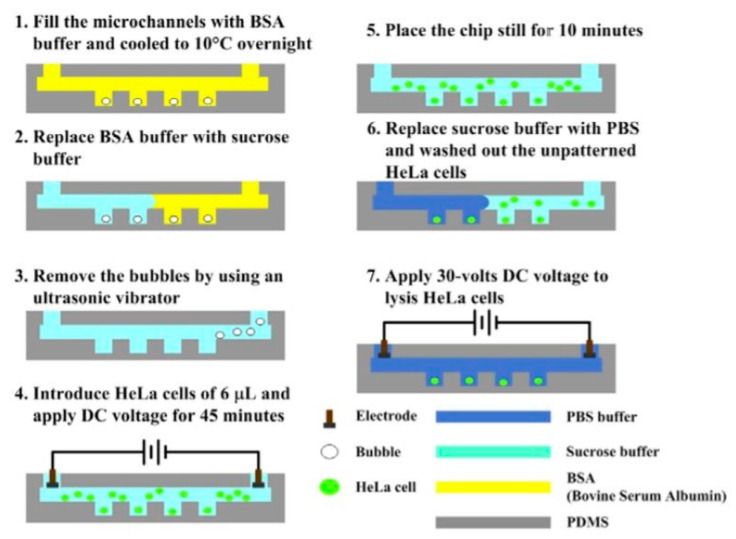
Experimental procedures for cell patterning utilized in [69].

Church et al. demonstrated a microfluidic platform, which first successfully concentrated red blood cells in a single constriction microchannel, via a DC-based electric field [68]. Cell lysis was then performed utilizing an AC electric field in this area. In these processes, the DC field drove the cell sample and controlled the cell traveling speed through the channel, whereas the AC field reached a field magnitude and gradient in the constriction region for the successful lysing. Further, both trapping and lysing have been used in conjunction to implement a selective enrichment and isolation of leukemia cells from red blood cells in the constriction microchannel. A novel method utilizing microwells in combination with an electrical field for the lysis of single-cells (HeLa cells) was developed by Jen et al. in [69]. With the help of an electroosmotic-driven flow, single-cells were trapped within arrays of 30 µm-diameter microwells–Figure 18. The highest cellular occupancy in the microwells (82.4%) occurred when the lowest voltage of 5 V was applied. When the voltage was increased to 15 V, the cellular occupancy in the microwells dropped to 64.3%. The results of the electric lysis at the single-cell level indicate that the cells were gradually lysed when a DC voltage of 30 V was applied; the cells were fully lysed after 25 s.

Similar results were observed by Ramadan et al. in [70]. The group developed a microfluidic chip for the electrical cell lysis (human white blood cells and murine clonal cells) and cell and bead trapping by dielectrophoresis (DEP). The electric field was generated by applying a sinusoidal voltage with different amplitudes of up to 10 V. The results showed that increasing the flow rate reduces each cell’s accumulated exposure to the electric field, which resulted in an overall decrease in the lysis rate. For example, lysis rates of MN9D cells reduced from 82% to 45% as the flow rates increased from 30 to 50 µL min^−1^, under the same electrical conditions. The highest lysis rate occurred for an applied voltage of 10 V, voltages above this value caused bubbles due to electrolysis. As mentioned above the lysis of bacterial cells could be a challenge due to the different structures of their cell membranes. Bao et al. investigated the electrical lysis of bacterial cells on a microfluidic chip [71]. Microscale silica beads (4.8 µm in diameter) were packed in a microfluidic channel, providing a microscale matrix that filtered *E. coli* cells in the solution. Subsequent electrical pulses rapidly lysed the cells and released intracellular proteins. The fluorescent protein (GFP) released by *E. coli* cells was detected when the field intensity was higher than 10 Vm^−1^ downstream of the channel. Such release was mostly finished within the first electrical pulse when the field intensity was at 12.5 V m^−1^. It needs to be noted that the field intensities described above were calculated based on the channel length and the applied voltage. The actual field intensity inside the bead array should have been higher.

**Figure 19:**
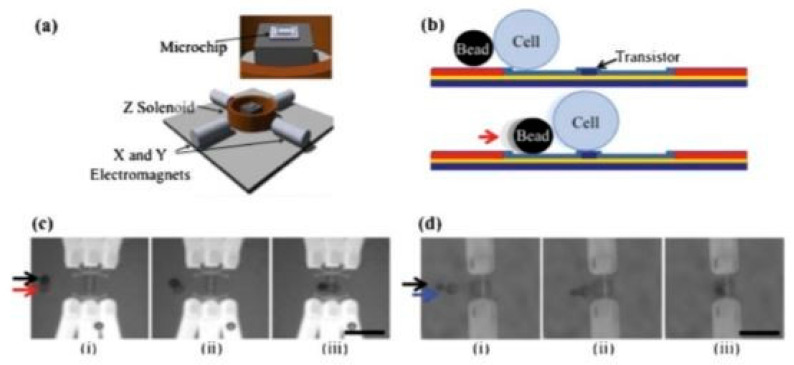
(**a**) Schematic of the magnetic bead setup utilized in [72]. The electromagnets generate a magnetic field, which rolls magnetic beads across the chip surface. (**b**) The beads are used to push the cells into position above the transistor. (**c**) An MCF-7 cell (red arrow) is moved from left to right by a bead (black arrow) to a position over a set of nanowires. (**d**) An HT-29 cell (blue arrow) is moved from left to right by a bead (black arrow) and positioned over a nanoribbon. Adapted with permission from [72]. Copyright 2013 Royal Society of Chemistry.

To further position and lyse individual cells, Jokilaakso et al. reported a new method to perform single-cell electroporation using silicon nanowires [72]. Utilizing magnetic beads and an external magnetic field, cells (HT-29 cancer cells) were easily manipulated and positioned above a field-effect transistor by regulating the frequency and strength of the rotating magnetic field. After applying a 10 MHz and 600 mV voltage between the shortened source-drain and the back gate of the transistor, cells were gently and specifically lysed in 2 ms. Figure 19 shows the working principle of the device.

**Figure 20:**
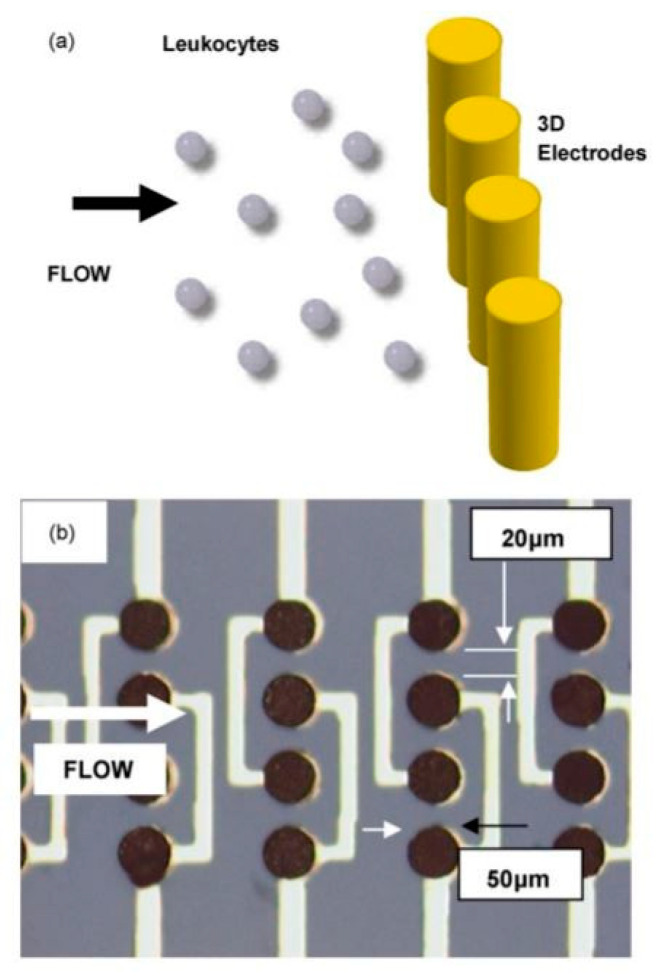
Three-dimensional cylindrical electrodes utilized in [75]. (**a**) Sketch of leukocytes approaching a singe row of electrode. The height of each electrode is 50 m. (**b**) The diameter of each electrode is 50 µm with spacing between them 20 µm. The height of each electrode is 50 µm. Adapted with permission from [75]. Copyright 2006 Elsevier.

To explore the effect of osmotic conditions and frequency on electroporation lysis, Hung et al. designed a microfluidic electroporation device to lyse a single cell and stretch its DNA [73]. Different hypotonic environments and electric fields were applied to investigate their effects on cell lysis. It was found that the most favorable conditions for lysis and DNA release are a hypotonic solution of 75 mM glucose solution, an AC voltage of 100 V, and a frequency of 1 kHz Although two-dimensional (2D) planar electrodes are widely utilized in the vast majority of micro-electroporation chips, they suffer from some major limitations. For example, since the cell size (10–20 µm) is typically much larger than the depth of the surface electrodes (<1 µm), the cell membrane tends to be exposed to a non-uniform electric field during the electroporation process. Three-dimensional (3D) electrodes have been used to address these challenges despite their complexity in manufacturing [74]. Lu et al. compared 3D electrodes to a 2D electrode pair of similar size [75]. The results revealed that 3D cylindrical Figure 20 electrodes had higher electroporation efficiency than 2D planar electrodes. Multiple pores could be generated in the cell membrane with 3D electroporation while only a single pore is generated with 2D electrodes.

**Figure 21:**
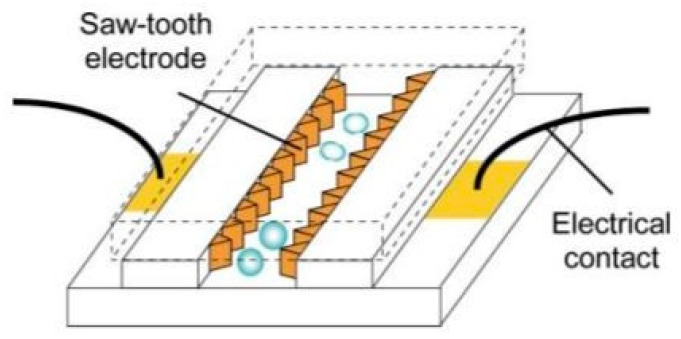
Schematics of a micro electroporation device with only one set of electrodes for cell lysis utilized in [76]. Adapted with permission from [76]. Copyright 2006 Royal Society of Chemistry.

In [76], a microfluidic device for the electroporation of mammalian cells was developed utilizing 3D electrodes. Figure 21 shows a schematic of the device. With the help of an AC-field (8.5 V, 10 kHz) the microdevice was effective in lysing cells while operating at more advantageous conditions such as small voltages, continuous flow, small sample volume, and negligible heating in comparison to 2D-electrodes. An efficiency rate of 83% was achieved. Similar results, showing the better lysing efficiency of 3D electrodes were shown in [77,78,79,80].

**Figure 22:**
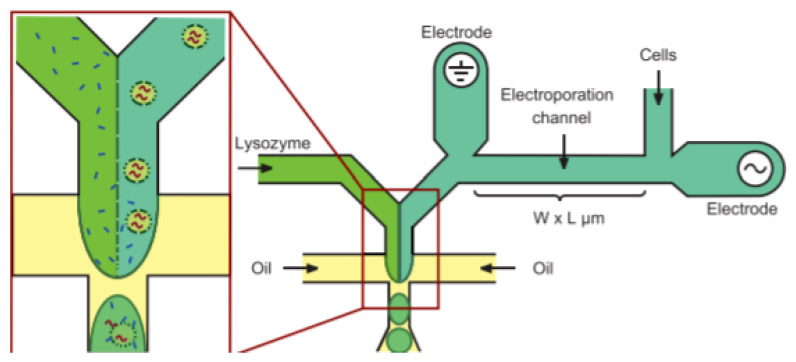
Schematic of the electrical lysis part and co-flow droplet generation part of the microfluidic device utilized in [74]. Adapted with permission from [74]. Copyright 2016 AIP Publishing.

Similar to chemical lysis techniques, droplets are also utilized with electrical lysis methods for higher efficiency of the process. Delange et al. proposed a microfluidic device, where cells were exposed to an electric field immediately before encapsulation in droplets [74]. The group included lysozyme, an enzyme that digests bacterial cell walls, via a second channel. As a cell passed through the device, as shown in Figure 22, it first flowed through the electric field channel, where the lysis was accomplished. Besides, the Péclet number (Pe) relating the ratio of advective to diffusive transport [81] was about 10,000, indicating that cells remained localized in their streamlines, as they traveled through the electrification channel. To investigate the ability of the device to lyse bacterial cells, *E. coli* cells were also utilized. When no field was applied, 99% of the cells remained unlysed. By contrast, when increasing the electric field to 22. 4 · 104 V/m roughly half the cells were lysed, while, 70% were lysed. Other studies combining droplet-microfluidics with the electroporation method are described in [82,83,84,85,86].

In contrast to conventional electrical techniques, where larger cells always lyse preferentially to smaller cells, Kremer et al. developed a novel method that enables shape-selectivity in such a way that cells with a different geometry will preferentially lyse from within a mixture of cell types [87]. To achieve this, the authors utilized the form of the cell to enhance a non-uniformity in the electric field. One of the electrodes in the system was a semiconductor, thus allowing cells close to this to affect the amount of the field within this semiconductor, and therefore changing the electrical potential at the liquid interface. This “electrical shadow” cast by a cell onto a semiconductor surface created a locally enhanced transmembrane field gradient, thus leading to poration and subsequent lysis. This effect was influenced also by the shape of the cell, providing a method for selectively lysing different types of cells.

### 2.4. Laser Lysis

**Figure 23:**
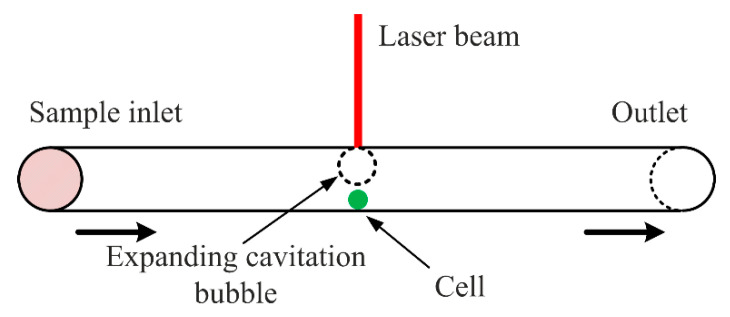
Schematic representation of the working mechanism of the laser lysis in microfluidics.

Laser microsurgery has demonstrated that cell membranes could be locally destroyed while keeping the interior of the cell undamaged [88]. Laser lysis involves the utilization of the fluid motion produced by a focused laser to rupture the cell membrane. When the laser pulse is focused at the buffer interface of a cell solution, it produces a localized cavitation bubble. The rupture of the cell membrane occurs after the expansion and the subsequent collapse of the bubble together with the induced fluid dynamic forces [6]. Figure 23 shows this mechanism.

By utilizing a time-resolved imaging technique and mechanical analysis, Rau et al. analyzed the lysis physics and kinetics behind this mechanism, the relationship between cell lysis and deformation of the created bubble [89]. After observation of the whole process and calculation of the hydrodynamic forces, it was found that the site of bubble formation determines the starting point of the lysis. Fluid flow during the bubble expansion is responsible for the cell lysis when the formation point is close to the cells. However, when the laser is away from cells (about 30 µm or above), cell lysis will start due to the shock wave induced by the bubble collapse (over 30 µs after the pulse). Similar results were shown in [90] and [91].

Wan et al. utilized an ultraviolet (UV) light array combined with titanium oxide particles [92]. When the titanium oxides were excited with the UV light array, electrons in the valence band were excited to conduction band, which results in electron-hole pairs. In an aqueous environment, these electron-hole pairs react with the surrounding molecules and generate free radicals such (OH, O, or O_2_^−^). These reacted with the cell membrane and lysed the cells (*E. coli*). The main disadvantage of this technique was that the high time required to lyse the cells (45 min). Huang et al. presented a microfluidic platform featuring optically-induced cell lysis (OICL) for nucleus extraction and collection [93]. The efficiency of cell membrane lysis and the nucleus separation were measured to be around 80.9%, respectively, leading to an overall nucleus extraction efficiency of about 60%. In the cell lysis zone, multiple light spots were projected from a digital projector a non-uniform electrical field was generated accordingly to create a required transmembrane potential across the cell membrane. The light intensity of these virtual electrodes was 3.2 Wcm^−2^ (white light), which allowed the membranes to be lysed without disruption of the nuclei. It has been also found that when laser lysis is incorporated with polydimethylsiloxane (PDMS) microchannel, the efficiency of the lysis process decreased [89]. A possible explanation may be the deformation of PDMS walls, which dissipates the mechanical energy from the bubble collapse; therefore, higher energy was required for the whole process.

### 2.5. Thermal Lysis

**Figure 24:**
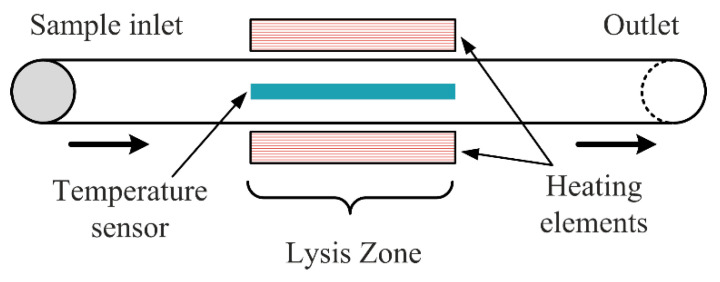
Thermal lysis in a microfluidic device.

Thermal lysis involves the usage of high temperature to denature the proteins within cell membranes, thus damaging the cells to access intracellular components. Typically, temperature sensors should be integrated into the channels to control the local temperature precisely, not to damage the cell proteins. Figure 24 shows a schematic generalization of the method. Most of the utilized thermal lysis methods are performed by ohmic heating, which consumes low power and is easily miniaturized for microfluidics chips [94].

**Figure 25:**
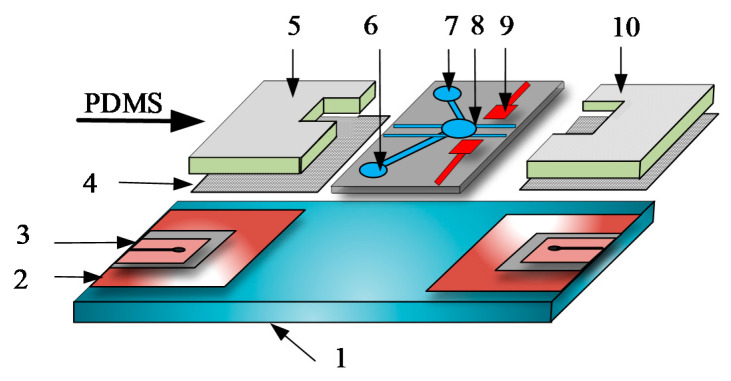
Schematic representation of the integrated microfluidic system (adopted from [95]). The microelectrodes deposited on the lower substrate act as driving elements, while the ones on the shielding channel are for the mixing of samples. **1**–glass substrate, **2**–micro heater, **3**–integrated temperature sensor, **4**–cover slide, **5**–micro lysis reactor, **6**–PCR reagent injection, **7**–primer injection, **8**–mixture reservoir, **9**–mixer, **10**–micro PCR camber.

Chia-Yen Lee et al. reported, for example, an automated microfluidic device for DNA amplification, using micro heaters and temperature sensors to regulate and measure the temperature inside the lysis chamber, as shown in Figure 25 (5) [95]. Based on this configuration, cells could be lysed within 2 min at a constant temperature of 95 °C. Extracted DNA samples, primers, and reagents are then driven electroosmotically into a micromixer (9) where they are mixed. The homogeneous mixture is then thermally cycled in a micro-PCR (10) (polymerase chain reaction) chamber to perform the DNA amplification. Micro-heaters (2), micro temperature sensors (3), and microelectrodes are deposited on the lower glass (1) substrate. The sensors and micro-heaters were located within the PCR reaction chamber itself in order to improve the temperature measurement capabilities. For this reason, it is showed that the proposed device can automate the sample pretreatment operation for DNA amplification.

Packard et al. demonstrated detergent-free, heat-only lysis in a single-step flow-through manner, on a microfluidic chip [96]. *E. coli* cells were utilized; the results of the lysis process were assessed by measurement of membrane compromise, protein release, and DNA release. Cells were lysed via a multi-turn serpentine microchannel. The channels were sealed by anodic bonding (350 °C, constant voltage −900 V, 5 min) to borosilicate glass. Significant cell membrane permeabilization, as indicated by increased ratios of red to green signal, (summed over the entire image area) was measured at 90 °C for residence times as short as 3.75 s. Results comparable to standard lysis techniques were achievable at temperatures greater than 65 °C and heating durations between 1 and 60 s. To further perform localized thermal lysis, Natalya Privorotskaya et al. developed a microcantilever device for heating cells [97]. The microcantilever heaters were coated with a layer of 100 nm thick electrically insulating ultrananocrystalline diamond (UNCD). When cells were immobilized on the microcantilever, the heaters provided local resistive heating to high temperatures, thus completing lysis within 30 s in a localized region. The device could effectively lyse mammalian and bacteria cells, but it does not rupture the cell walls of some intractable microorganisms, according to the results. Burklund et al. demonstrated a microfluidic device, which enabled highly controlled, on-chip heating for the lysis of bacteria cells [98]. The presented chip utilized an AC external magnetic field (AMF). Due to their specific absorption rate (SAR), approaching the theoretical limit when exposed to a clinically relevant alternating magnetic field (AMF), iron oxide nanoparticles were selected as a magnetic component of the polymeric material. These magnetic nanoparticles were spiked into a polydimethylsiloxane (PDMS), increasing the heat efficiency of the system. Following exposure to an AMF, bacteria were thermally lysed, enabling additional on-chip and/or downstream nucleic acid amplification and analysis. Reaching temperatures of up to 110 °C, the device showed a lysis efficiency of 90% for 60 s exposure time. Similar devices, utilizing the heat properties of nanoparticles for the thermal lysis of cells are described in [99,100].

**Figure 26:**
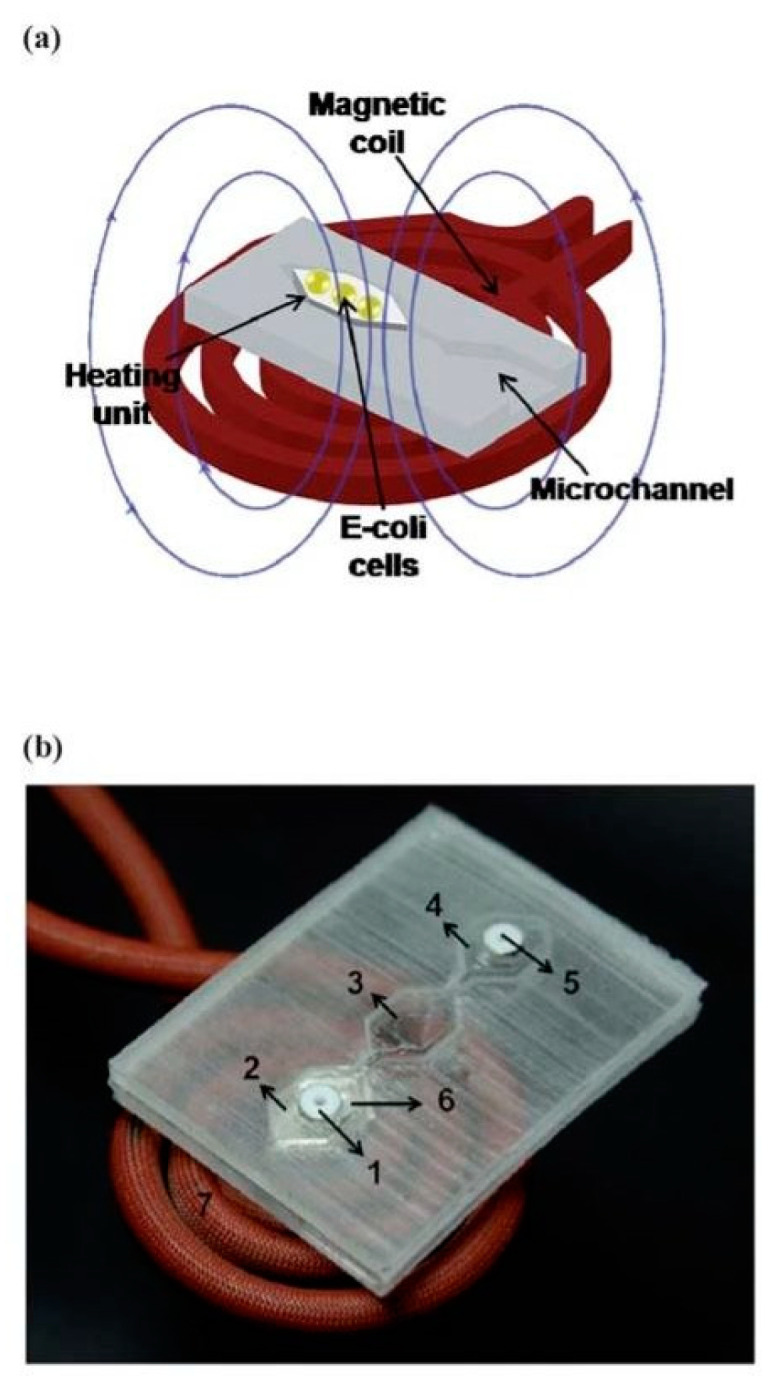
Representation (**a**) and a photograph (**b**) of the integrated microfluidic system utilized in [101]. A metal heating unit with a hexagonal shape was embedded in a bottom layer of polydimethylsiloxane and a hexagonal metal heating unit was heated at a specified magnetic field. Adapted with permission from [101]. Copyright 2010 Royal Society of Chemistry.

Another device making use of an alternating magnetic field for cell lysis was presented by Baek et al. [101]. After comparing the thermal responses of nickel, iron, and copper heating, a nickel structure was selected as a heating element, because of its faster thermal response and relatively small geometric influence. Wireless induction of heating from the magnetic field on the hexagonal-shaped metal was embedded in the bottom layer of the microchannel, as shown in Figure 26. The amount of protein extracted was proportional to the applied magnetic field (ranging from 110 to 170 mT for all metals), with almost 80% for the nickel element. It was shown that the device represented a reasonable alternative to commercial RNA extraction methods.

### 2.6. Acoustic and Electrochemical Methods

**Figure 27:**
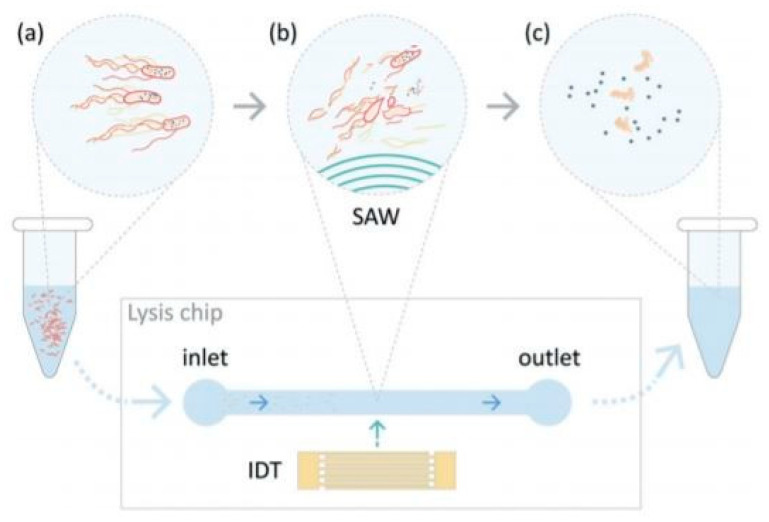
Overview of the TSAW lysis device utilized in [104]. The lysis chip consists of a PDMS microchannel plasma-bonded to a lithium niobate (LiNbO3) substrate with an interdigitated transducer (IDT) adjacent to the microchannel to generate traveling surface acoustic waves. Bacteria suspension was flown through the microchannel (**a**) the bacteria suspension was exposed to a TSAW field (**b**) and the lysate for further analysis were collected (**c**). Adapted with permission from [104]. Copyright 2019 Royal Society of Chemistry.

The spectrum of existing methods for cell lysis does not summarize in just five categories. In addition to all the devices presented above, there are still some lysis methods that are hard to categorize. For example, a surface acoustic wave (SAW) is a nanometer-order amplitude-traveling wave that propagates on the surface of a piezoelectric crystal substrate [102,103]. When supplied with an electrical signal, the interdigital transducer (IDT) produces a periodic strain on the surface resulting in an acoustic wave propagating away from it. This device can effectively lyse cells without the use of lysis buffer or complex operations, so it has good potential for further integration into on-chip technologies. This technique was utilized by Lu et al. [104]. The team characterized the effects of the SAW on *E. coli* by measuring the viability of cells exposed to the acoustic waves Figure 27 and find that about 90% are dead, about 20% of the intracellular material (nucleic acids and proteins) were successfully recovered.

**Figure 28:**
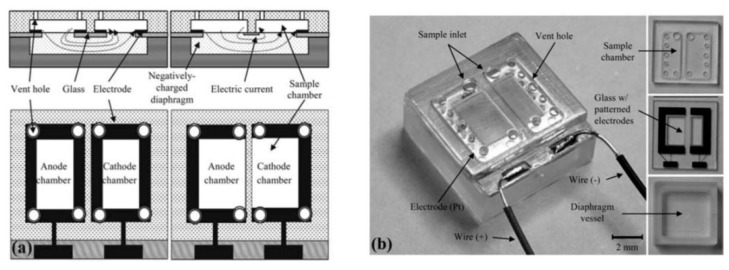
Design of the electrochemical cell lysis devices utilized in [109]. (**a**) Cross-sectional and top-down views of the micro-devices. Cathode and anode chambers were separated by an ion exchangeable polymer diaphragm. (**b**) Photographs of the same microdevice. Adapted with permission from [109]. Copyright 2010 Royal Society of Chemistry.

Reboud et al. [105] developed a microfluidic chip to detect the rodent malaria parasite Plasmodium berghei in blood. They used the SAW method to lyse red blood cells and parasitic cells in a drop of blood. The reported lysis efficiency was more than 99.8%. Other papers describing this technique are shown in [106,107,108]. Lee et al. presented an on-chip device for the electrochemical lysis of cells (*E. coli*, *Pseudomonas putida*, *Staphylococcus epidermidis*, and *Streptococcus mutans*) [109]. It utilizes the electrolysis of saline solution to generate hydroxide ions (OH^−^) at the cathode as alkaline lytic agents. Cathode and anode chambers were separated by a negatively charged ion exchangeable polymer diaphragm to maintain the high pH level for efficient cell lysis in the cathode chamber, as shown in Figure 28. Real-time PCR analysis showed that the device could lyse both Gram-positive and Gram-negative bacterial cells with higher efficiency than other common methods. The same technique was utilized in [110].

If the salt concentration in the surrounding of a cell is changed so that there is a concentration difference between the inside and outside of the cell, the cell membrane becomes permeable to water due to osmosis [2,111]. If the concentration of salt is lower in the surrounding solution, water enters the cell and the cell swells up and subsequently bursts. It was shown that when the cells were pretreated with divalent cation (Ca^+^ or Mg^+^) the efficiency of the osmotic shock method could be improved to 75%.

## 3. Discussion and Conclusions

This paper reviews applications of microfluidics devices for cell lysis and categorizes them into six major groups. It expands upon the approaches from the last couple of years and adds more details to the techniques than other review papers. Most of these existing microfluidic devices for cell lysis are developed as standalone units. Integrating the cell lysis microfluidic part with other function blocks, to make a complete diagnostic system is no doubt of great importance and challenge as well. Thus, it is crucial to understand the advantages and disadvantages of every method. It is possible for example to make use of general criteria for comparing the individual methods, such as cost, efficiency, lysis time, and technical difficulty of the process. Chemical lysis being, for example, according to most of the studies considered in this article, the cheapest method in terms of the price of the required instruments, easiest at handling them, and very efficient (reflected by the number of broken cells). However, in most cases, this is compensated by the fact that much more time is needed for the cells to lyse than the other methods. The effect of the buffers on the cell proteins is in most cases not so harmless so that an additional step is needed to filter out unwanted substances [97]. Chemistry needs also to be modified for different cell types. Highly efficient but also complex and expensive systems, on the other hand, are the main characteristics of laser lysis. For this reason, this method is utilized mainly for research purposes today. Thermal lysis is reported to be one of the earliest methods for cell lysis [1]. In general, thermal lysis is effective in a microfluidic platform, according to [112] however, these devices are not suitable for sample preparation where the sample is of a large volume, and cells have to be lysed from a continuous flow. The method requires also very precise handling to avoid lateral effects such as high-temperature development, which could change the consistency of the needed cell materials. Mechanical lysis, as discussed, covers a wide range of physical effects (from centrifugal effects, magnetization lysis to pressure-based membranes) and, thus, offers a universal possibility for the lysis of different cell types. On the other hand, the fabrication of these mechanical devices may be very complex as well as expensive. Collecting the target materials from a complex mixture may also bring some problems. Electrical methods offer a simple, fast, and reagent-less lysis procedure to lyse various kinds of cells being also suitable for selective lysis. The requirement of high voltages is in some cases a problem, due to heat generation and formation of a bubble, it can be however overcome by decreasing the gap between electrodes and a suitable design [62]. Future works could also concentrate on the development of cheap and reliable electrodes, similar to the one described in [113]. Summarized comparison of these five methods is presented in Table 1.

The dream scenario for the microfluidic world would be the effective integration and total on-chip single-cell analysis. For this reason, it is important, that cell lysis, being an important part of the workflow, does not undermine the performance of other steps [114]. The design simplicity of the lysis unit is desirable. There should be no additional treatments to enable downstream reactions. Techniques with high lysis speed are favored to minimize sample dilution and ensuring an effective coupling between functional units. On the other hand, the effectiveness of the lysis process should be the same for different cell types.

## Figures and Tables

**Table 1:** Summarized comparison of the described methods.

Lysis Type	Efficiency	Lysis Time	Technical Difficulty	Cost
Chemical	High	Slow/Moderate	Low	Low
Mechanical	Medium	Moderate	Medium	Medium
Electrical methods	High	Fast	High	High
Thermal	Medium	Moderate	Medium	Medium
Laser	High	Very Fast	Very High	Very High
Acoustic	High	Very Fast	High	High
Electrochemical	Medium	Moderate	High	Moderate

## Sample Availability

Samples of the compounds are available from the authors.

## Abbreviations

The following abbreviations are used in this manuscript:

CFD	Computational fluid dynamics
SDS	Sodium dodecyl sulfate
DNA	Deoxyribonucleic acid
PCR	Polymerase chain reaction
CCO	Cell Crossover
*E. coli*	*Escherichia coli*
